# The genome sequence of the Pink Pigeon, *Nesoenas mayeri *(Prévost, 1843)

**DOI:** 10.12688/wellcomeopenres.22471.1

**Published:** 2024-06-24

**Authors:** Hernán E. Morales, Cock van Oosterhout, Harriet Whitford, Vikash Tatayah, Kevin Ruhomaun, Jim J. Groombridge, M. Thomas P. Gilbert, Wellcome Sanger Institute Tree of Life Management, Samples and Laboratory team, Wellcome Sanger Institute Scientific Operations: Sequencing Operations, Wellcome Sanger Institute Tree of Life Core Informatics team, Tree of Life Core Informatics collective

**Affiliations:** 1 Globe Institute, University of Copenhagen, Copenhagen, Denmark; 2 Centre for Evolutionary Hologenomics, University of Copenhagen, Copenhagen, Denmark; 3 School of Environmental Sciences, University of East Anglia, Norwich, England, UK; 4 Durrell Wildlife Conservation Trust UK, Trinity, Trinity, Jersey; 5 Mauritian Wildlife Foundation, Vacoas, Mauritius; 6 National Parks and Conservation Service (Government of Mauritius), Reduit, Mauritius; 7 Durrell Institute of Conservation and Ecology, Division of Human and Social Sciences, University of Kent, Canterbury, England, UK

**Keywords:** Nesoenas mayeri, Pink Pigeon, genome sequence, chromosomal, Columbiformes

## Abstract

We present a genome assembly from an individual female *Nesoenas mayeri* (the Pink Pigeon; Chordata; Aves; Columbiformes; Columbidae). The genome sequence is 1,183.3 megabases in span. Most of the assembly is scaffolded into 40 chromosomal pseudomolecules, including the Z and W sex chromosomes. The mitochondrial genome has also been assembled and is 16.97 kilobases in length. Gene annotation of this assembly on Ensembl identified 16,730 protein coding genes.

## Species taxonomy

Eukaryota; Opisthokonta; Metazoa; Eumetazoa; Bilateria; Deuterostomia; Chordata; Craniata; Vertebrata; Gnathostomata; Teleostomi; Euteleostomi; Sarcopterygii; Dipnotetrapodomorpha; Tetrapoda; Amniota; Sauropsida; Sauria; Archelosauria; Archosauria; Dinosauria; Saurischia; Theropoda; Coelurosauria; Aves; Neognathae; Columbiformes; Columbidae; *Nesoenas*; *Nesoenas mayeri* (Prévost, 1843) (NCBI:txid187126).

## Background

**Figure 1.**
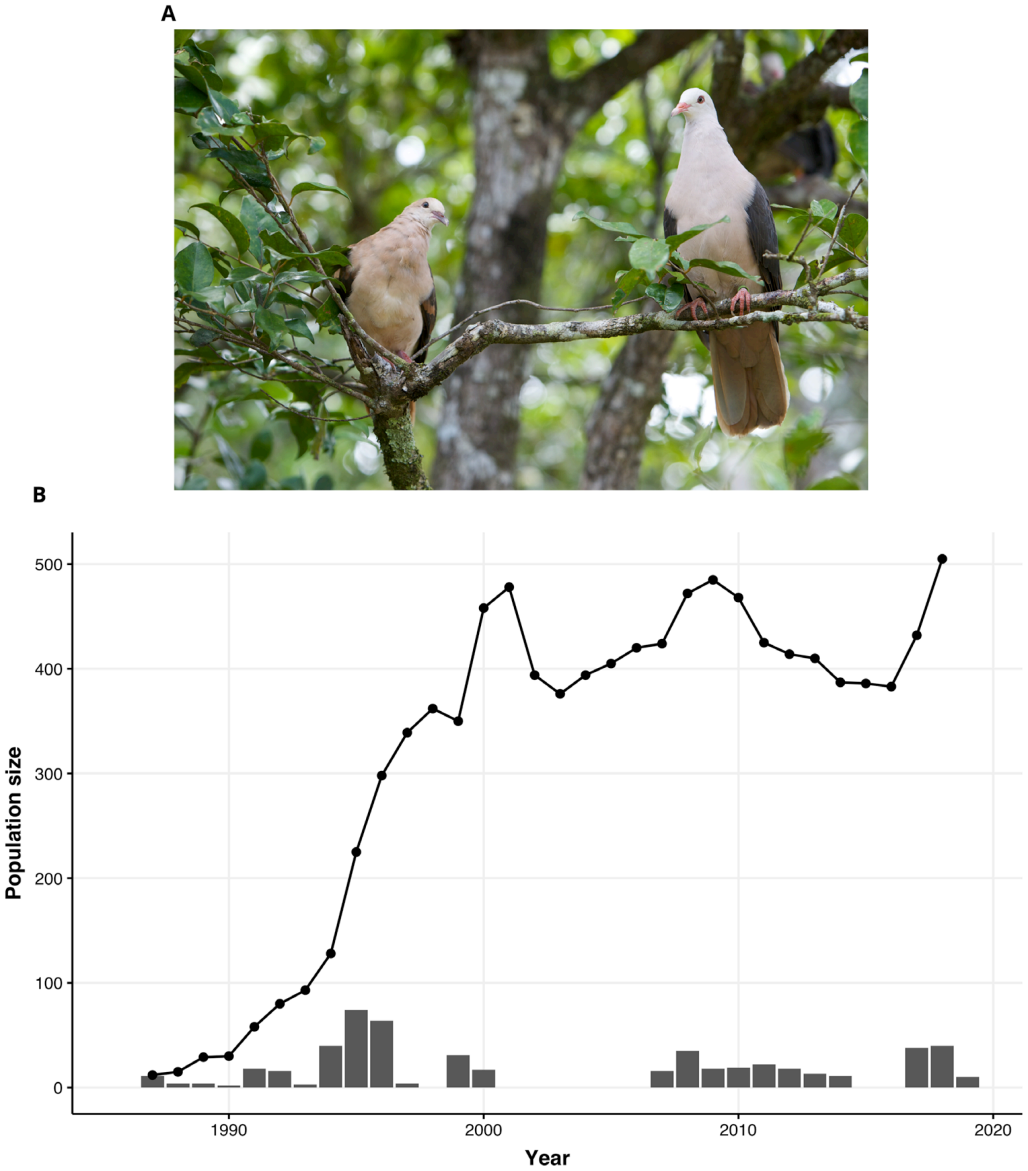
The fall and rise of the Pink Pigeon. ( **A**) A Pink Pigeon ( *Nesoenas mayeri;* photo credit Gregory Guida) ( **B**) Demographic trajectory (black line and dots) derived from field monitoring, of the free-living Mauritius Pink Pigeon population over time (bottleneck and recovery), and the bars represent the number of captive-breed individuals released into the free-living population.

The Pink Pigeon ( *Nesoenas mayeri*) is an endemic species of Mauritius distinguished by its soft pinkish-grey feathers and bright pink legs ( Figure 1A). This bird primarily feeds on leaves, seeds, and fruits from native and non-native plants. Pink Pigeons nest on branches, laying clutches of 1 to 2 eggs mainly during the breeding season from September to January. The Pink Pigeon suffered a severe population size decline due to habitat loss and invasive species ( Jackson *et al.*, 2022; Jones, 2013; Pinto *et al.*, 2024). By 1990, the free-living population consisted of only circa 10 individuals ( Jones, 2013; Jones & Swinnerton, 1997). Prior to this population bottleneck, 12 individuals were taken from the last free-living population to establish a captive breeding population at the Gerald Durrell Endemic Wildlife Sanctuary (GDEWS) in Mauritius between 1976 to 1981. This captive-bred population also helped to establish the zoo populations of Pink Pigeons in Europe and America. The population at the GDEWS has also contributed to demographic rescue of the free-living population ( Jackson *et al*. 2022; Jones, 2013). The current free-living population in Mauritius is estimated to comprise of ~488 adult birds in Mauritius ( Figure 1B) ( BirdLife International, 2021).

The species was assessed as Critically Endangered (between 1994–2000) in the IUCN Red List, and downlisted to Endangered in 2000, and then to Vulnerable in 2018. According to the IUCN’s Green Status assessment ( Tatayah, 2021), the Species Recovery Score equals 17% (Critically Depleted), which is low due to massive forest loss. However, the Pink Pigeon has a High Conservation Legacy, given that without past conservation action, the species would almost certainly be extinct today assessment ( Tatayah, 2021). Genomic-based analysis and computer simulation studies indicate that without genetic rescue, the species is likely to go extinct within the next 50 to 100 years ( Jackson *et al.*, 2022).

Quantitative genetic and conservation genomic analyses show that the Pink Pigeon suffers from severe genomic erosion and a considerable ‘drift debt’ ( Pinto *et al.*, 2024). The species possesses a high genetic load of deleterious mutations, which is estimated to amount to 15 lethal equivalents ( Jackson *et al*., 2022). Hence, continued genetic drift and inbreeding are predicted to result in severe inbreeding depression by increasing the realised load of deleterious mutations ( Bertorelle *et al.*, 2022; Dussex *et al.*, 2023). Recent genomics research on the Pink Pigeon shows that genomics-informed captive breeding can reduce the realised load by selecting optimal mate-pairs for captive breeding ( Speak *et al.*, 2024). In addition, to improve the long-term viability of the species, three captive-bred birds from the population in Jersey Zoo (British Channel Island) were transported to Mauritius in 2021. Furthermore, in collaboration with the National Parks and Conservation Service (NPCS) and the Mauritian Wildlife Foundation (MWF), a genomics-informed rescue programme is currently being established to inform future releases of captive-bred Pink Pigeons from Jersey Zoo and European zoos to Mauritius. Such genetic rescue is likely to increase diversity in the free-living population, and it will help mask the load of recessive deleterious mutations, thereby increasing fitness and population viability.

The comprehensive sample archive and profound understanding of the species’ ecology and its conservation legacy establish it as an exemplary system for studying conservation genomics. Currently, hundreds of whole genomes are being re-sequenced from historical (pre-1900), recent (1990–2000) and contemporary samples to uncover the genomic impacts and enduring consequences of the population's decline and revealing ways to optimize the long-term viability of the Pink Pigeon in Mauritius. This research efforts are part of a collaboration between several universities (University of Kent (UK), University of East Anglia (UK), University of Copenhagen (Denmark)), the Durrell Wildlife Conservation Trust (UK), Jersey Zoo, the Government of Mauritius’ National Parks and Conservation Service (NPCS) and the Mauritian Wildlife Foundation (MWF – conservation NGO, Mauritius). The conservation monitoring and management of the Pink Pigeon is done by the MWF in collaboration with the NPCS with guidance from the university partners; recent conservation actions have also been implemented by Ebony Forest Reserve (conservation group).

## Genome sequence report

The genome was sequenced from a female *Nesoenas mayeri* collected from Jersey Zoo, UK. A total of 32-fold coverage in Pacific Biosciences single-molecule HiFi long reads was generated. Primary assembly contigs were scaffolded with chromosome conformation Hi-C data. Manual assembly curation corrected 96 missing joins or mis-joins and removed 3 haplotypic duplications, reducing the scaffold number by 32.23%, and increasing the scaffold N50 by 10.48%.

**Figure 2.**
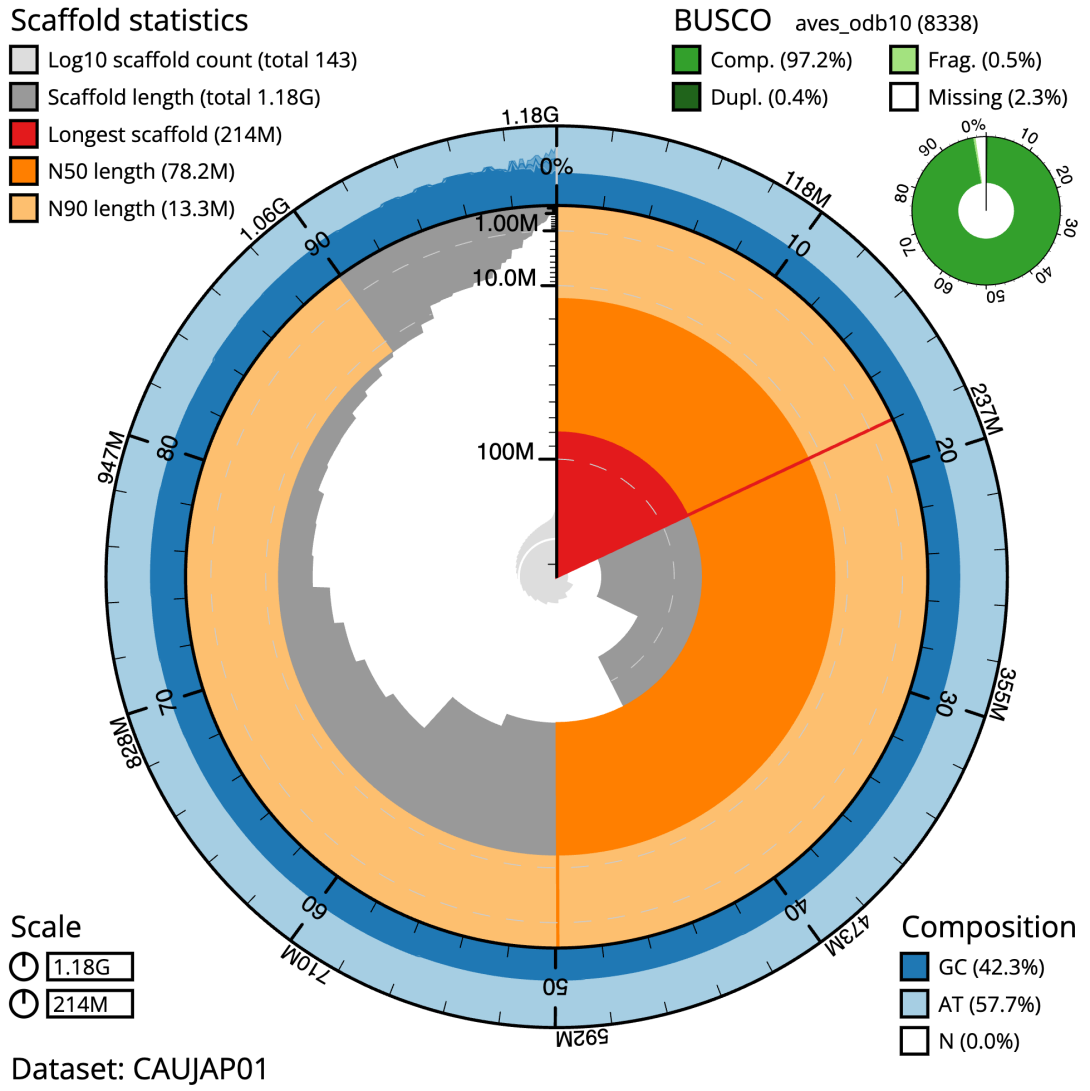
Genome assembly of *Nesoenas mayeri*, bNesMay2.1: metrics. The BlobToolKit snail plot shows N50 metrics and BUSCO gene completeness. The main plot is divided into 1,000 size-ordered bins around the circumference with each bin representing 0.1% of the 1,183,271,950 bp assembly. The distribution of scaffold lengths is shown in dark grey with the plot radius scaled to the longest scaffold present in the assembly (214,152,265 bp, shown in red). Orange and pale-orange arcs show the N50 and N90 scaffold lengths (78,184,682 and 13,300,769 bp), respectively. The pale grey spiral shows the cumulative scaffold count on a log scale with white scale lines showing successive orders of magnitude. The blue and pale-blue area around the outside of the plot shows the distribution of GC, AT and N percentages in the same bins as the inner plot. A summary of complete, fragmented, duplicated and missing BUSCO genes in the aves_odb10 set is shown in the top right. An interactive version of this figure is available at https://blobtoolkit.genomehubs.org/view/GCA_963082525.1/dataset/CAUJAP01/snail.

**Figure 3.**
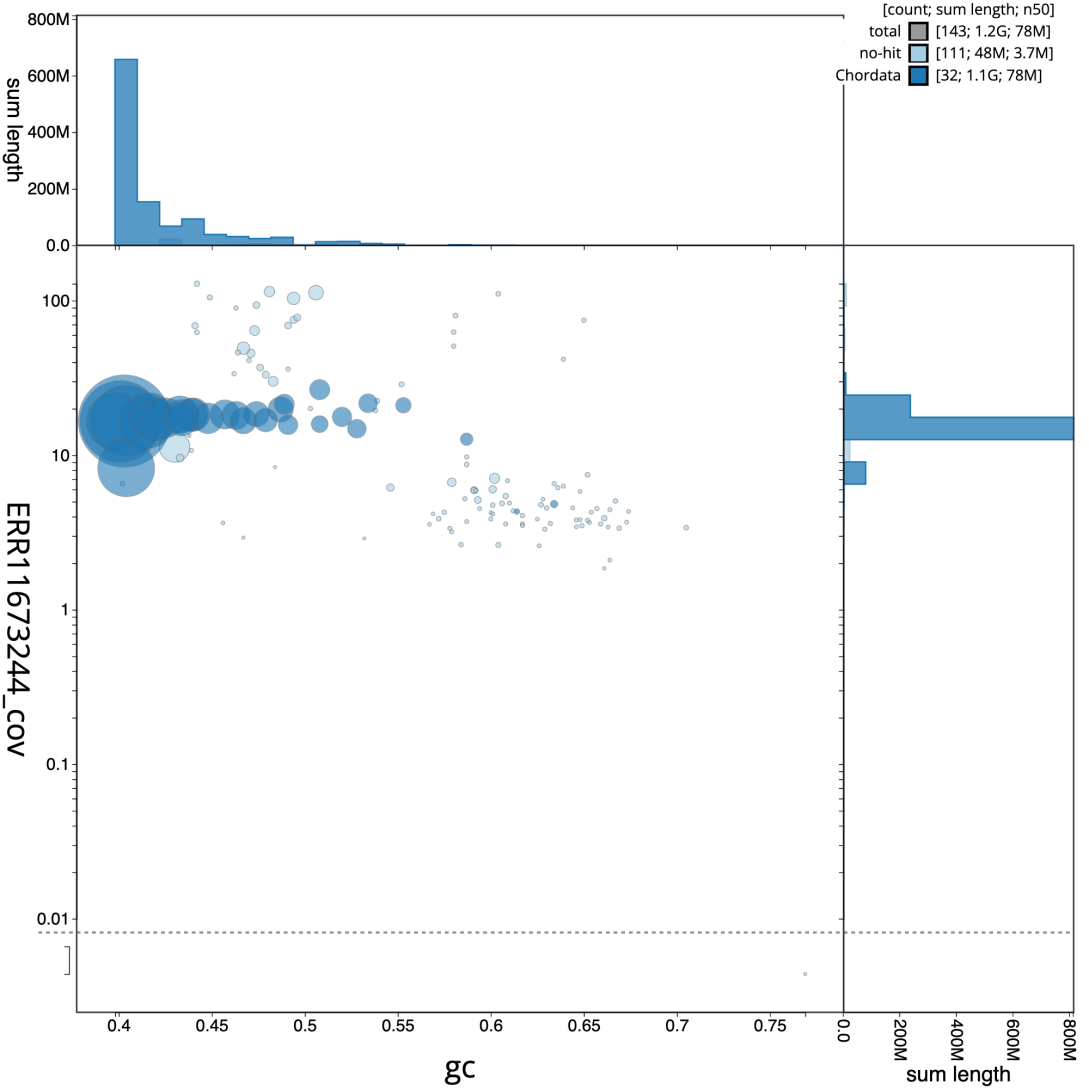
Genome assembly of *Nesoenas mayeri*, bNesMay2.1: BlobToolKit GC-coverage plot. Sequences are coloured by phylum. Circles are sized in proportion to sequence length. Histograms show the distribution of sequence length sum along each axis. An interactive version of this figure is available at https://blobtoolkit.genomehubs.org/view/GCA_963082525.1/dataset/CAUJAP01/blob.

**Figure 4.**
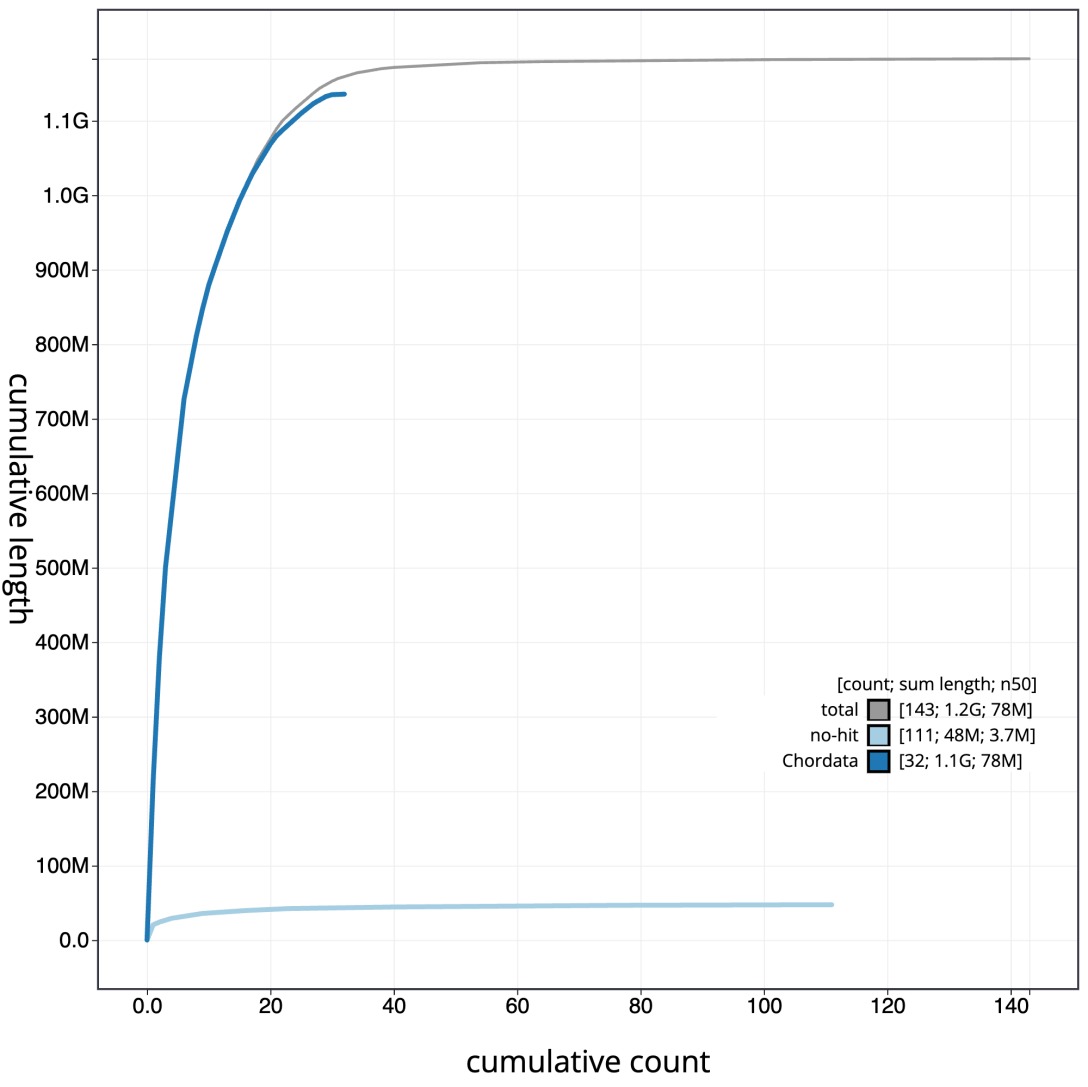
Genome assembly of *Nesoenas mayeri* bNesMay2.1: BlobToolKit cumulative sequence plot. The grey line shows cumulative length for all sequences. Coloured lines show cumulative lengths of sequences assigned to each phylum using the buscogenes taxrule. An interactive version of this figure is available at https://blobtoolkit.genomehubs.org/view/GCA_963082525.1/dataset/CAUJAP01/cumulative.

**Figure 5.**
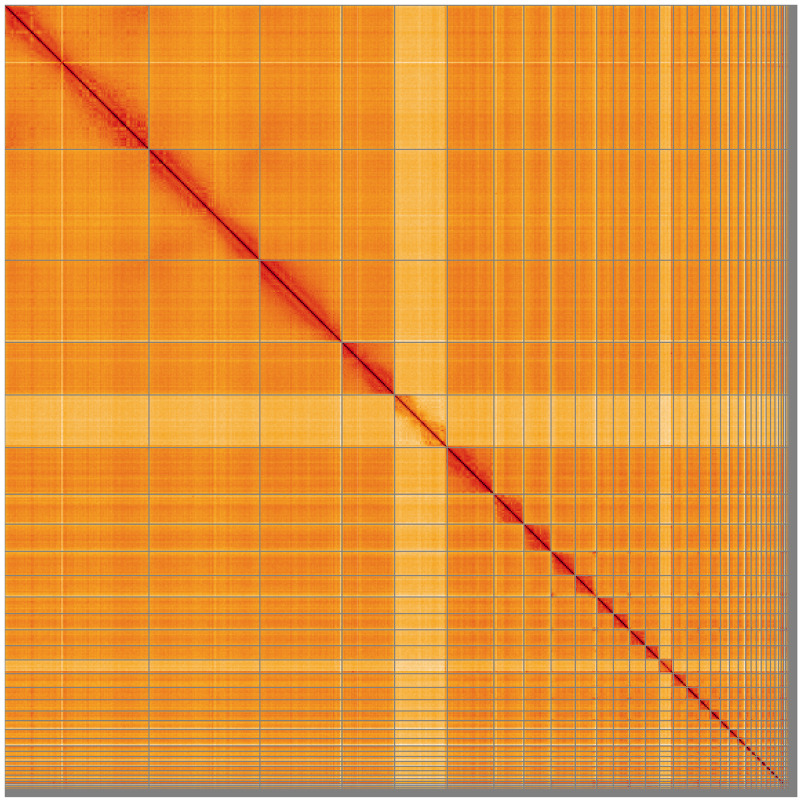
Genome assembly of *Nesoenas mayeri* bNesMay2.1: Hi-C contact map of the bNesMay2.1 assembly, visualised using HiGlass. Chromosomes are shown in order of size from left to right and top to bottom. An interactive version of this figure may be viewed at https://genome-note-higlass.tol.sanger.ac.uk/l/?d=U63e71O9TGGSWoX1VPKRfw.

The final assembly has a total length of 1,183.3 Mb in 142 sequence scaffolds with a scaffold N50 of 78.2 Mb ( Table 1). The snail plot in Figure 2 provides a summary of the assembly statistics, while the distribution of assembly scaffolds on GC proportion and coverage is shown in Figure 3. The cumulative assembly plot in Figure 4 shows curves for subsets of scaffolds assigned to different phyla. Most (98.45%) of the assembly sequence was assigned to 40 chromosomal-level scaffolds, representing 38 autosomes and the Z and W sex chromosomes. Chromosome-scale scaffolds confirmed by the Hi-C data are named in order of size ( Figure 5; Table 2). While not fully phased, the assembly deposited is of one haplotype. Contigs corresponding to the second haplotype have also been deposited. The mitochondrial genome was also assembled and can be found as a contig within the multifasta file of the genome submission.

**Table 1.** Genome data for *Nesoenas mayeri*, bNesMay2.1.

Project accession data		
Assembly identifier	bNesMay2.1	
Species	*Nesoenas mayeri*	
Specimen	bNesMay2	
NCBI taxonomy ID	187126	
BioProject	PRJEB64092	
BioSample ID	Genome sequencing: PacBio: SAMEA12922164	
Isolate information	bNesMay2, female: blood (genome sequence, Hi-C and RNA sequencing)	
Assembly metrics *		*Benchmark*
Consensus quality (QV)	62.6	*≥ 50*
*k*-mer completeness	100.0%	*≥ 95%*
BUSCO **	C:97.3%[S:96.9%,D:0.4%], F:0.5%,M:2.2%,n:8,338	*C ≥ 95%*
Percentage of assembly mapped to chromosomes	98.45%	*≥ 95%*
Sex chromosomes	ZW	*localised homologous pairs*
Organelles	Mitochondrial genome: 16.97 kb	*complete single alleles*
Raw data accessions		
PacificBiosciences Sequel IIe	ERR11673243, ERR11673244	
Hi-C Illumina	ERR11679408	
PolyA RNA-Seq Illumina	ERR11679409	
Genome assembly		
Assembly accession	GCA_963082525.1	
*Accession of alternate haplotype*	GCA_963082445.1	
Span (Mb)	1,183.3	
Number of contigs	652	
Contig N50 length (Mb)	4.8	
Number of scaffolds	142	
Scaffold N50 length (Mb)	78.2	
Longest scaffold (Mb)	214.15	
Genome annotation		
Number of protein-coding genes	16,730	
Number of non-coding genes	1,067	
Number of gene transcripts	27,410	

* Assembly metric benchmarks are adapted from column VGP-2020 of “Table 1: Proposed standards and metrics for defining genome assembly quality” from Rhie *et al.* (2021). ** BUSCO scores based on the vertebrata_odb10 BUSCO set using version 5.4.3. C = complete [S = single copy, D = duplicated], F = fragmented, M = missing, n = number of orthologues in comparison. A full set of BUSCO scores is available at https://blobtoolkit.genomehubs.org/view/GCA_963082525.1/dataset/CAUJAP01/busco.

**Table 2.** Chromosomal pseudomolecules in the genome assembly of *Nesoenas mayeri*, bNesMay2.

INSDC accession	Chromosome	Length (Mb)	GC%
OY720056.1	1	214.15	40.5
OY720057.1	2	164.7	40.0
OY720058.1	3	121.94	40.5
OY720059.1	4	78.2	40.0
OY720061.1	5	69.28	41.5
OY720062.1	6	44.46	41.5
OY720063.1	7	40.34	42.0
OY720064.1	8	36.08	42.5
OY720065.1	9	31.79	43.5
OY720066.1	10	24.72	44.0
OY720067.1	11	24.03	44.0
OY720068.1	12	23.58	43.5
OY720069.1	13	21.15	43.5
OY720071.1	14	20.36	45.0
OY720072.1	15	18.29	45.5
OY720073.1	16	16.72	46.5
OY720074.1	17	14.4	46.5
OY720075.1	18	13.3	48.5
OY720076.1	19	13.17	47.5
OY720077.1	20	11.03	48.0
OY720078.1	21	7.92	51.0
OY720079.1	22	7.77	49.0
OY720080.1	23	7.3	52.0
OY720081.1	24	7.27	49.0
OY720082.1	25	6.63	53.5
OY720083.1	26	6.53	53.0
OY720084.1	27	5.15	51.0
OY720085.1	28	4.18	55.5
OY720086.1	29	3.71	50.5
OY720087.1	30	2.43	58.5
OY720088.1	31	1.46	60.0
OY720089.1	32	0.87	58.0
OY720090.1	33	0.58	60.0
OY720091.1	34	0.56	54.5
OY720092.1	35	0.53	63.5
OY720093.1	36	0.41	59.0
OY720094.1	37	0.39	59.0
OY720095.1	38	0.33	59.0
OY720070.1	W	20.52	43.0
OY720060.1	Z	78.18	40.5
OY720096.1	MT	0.02	45.5

The estimated Quality Value (QV) of the final assembly is 62.6 with *k*-mer completeness of 100.0%, and the assembly has a BUSCO v completeness of 97.3% (single = 96.9%, duplicated = 0.4%), using the vertebrata_odb10 reference set ( *n* = 8,338).

Metadata for specimens, barcode results, spectra estimates, sequencing runs, contaminants and pre-curation assembly statistics are given at https://links.tol.sanger.ac.uk/species/187126.

## Genome annotation report

The *Nesoenas mayeri* genome assembly (GCA_963082525.1) was annotated at the European Bioinformatics Institute (EBI) on Ensembl Rapid Release. The resulting annotation includes 27,410 transcribed mRNAs from 16,730 protein-coding and 1,087 non-coding genes ( Table 1; https://rapid.ensembl.org/Nesoenas_mayeri_GCA_963082525.1/Info/Index).

## Methods

### Sample acquisition and nucleic acid extraction

A female *Nesoenas mayeri* (specimen ID SAN1100036, ToLID bNesMay2) was collected from Jersey Zoo, UK (latitude –2.08, longitude 49.23) on 2021-03-19. The bird was caught in the aviary, blood collected from the jugular vein, and the blood sample was frozen approximately 10 minutes later. The specimen was collected and identified by Harriet Whitford (Durrell Wildlife Conservation Trust).

**Sampling**: exampleThe workflow for high molecular weight (HMW) DNA extraction at the Wellcome Sanger Institute (WSI) Tree of Life Core Laboratory includes a sequence of core procedures: sample preparation; sample homogenisation, DNA extraction, fragmentation, and clean-up. The bNesMay2 sample was kept on dry ice ( Jay *et al.*, 2023). For sample homogenisation, blood was cryogenically disrupted using the Covaris cryoPREP ^®^ Automated Dry Pulverizer ( Narváez-Gómez *et al.*, 2023). HMW DNA was extracted using the manual Nucleated Blood Nanobind ^®^ protocol ( Denton *et al.*, 2023a). DNA was sheared into an average fragment size of 12–20 kb in a Megaruptor 3 system with speed setting 31 ( Bates *et al.*, 2023). Sheared DNA was purified by solid-phase reversible immobilisation ( Strickland *et al.*, 2023). The concentration of the sheared and purified DNA was assessed using a Nanodrop spectrophotometer and Qubit Fluorometer and Qubit dsDNA High Sensitivity Assay kit. Fragment size distribution was evaluated by running the sample on the FemtoPulse system.

RNA was extracted from blood tissue of bNesMay2 in the Tree of Life Laboratory at the WSI using the RNA Extraction: Automated MagMax™ *mir*Vana protocol ( do Amaral *et al.*, 2023). The RNA concentration was assessed using a Nanodrop spectrophotometer and a Qubit Fluorometer using the Qubit RNA Broad-Range Assay kit. Analysis of the integrity of the RNA was done using the Agilent RNA 6000 Pico Kit and Eukaryotic Total RNA assay.

Protocols developed by the WSI Tree of Life laboratory are publicly available on protocols.io ( Denton *et al.*, 2023b).

### Sequencing

Pacific Biosciences HiFi circular consensus DNA sequencing libraries were constructed according to the manufacturers’ instructions. Poly(A) RNA-Seq libraries were constructed using the NEB Ultra II RNA Library Prep kit. DNA and RNA sequencing was performed by the Scientific Operations core at the WSI on Pacific Biosciences Sequel IIe (HiFi) and Illumina NovaSeq 6000 (RNA-Seq) instruments. Hi-C data were also generated from the bNesMay2 blood sample using the Arima2 kit and sequenced on the Illumina NovaSeq 6000 instrument.

### Genome assembly and curation

Assembly was carried out with Hifiasm ( Cheng *et al.*, 2021) and haplotypic duplication was identified and removed with purge_dups ( Guan *et al.*, 2020). The assembly was then scaffolded with Hi-C data ( Rao *et al.*, 2014) using YaHS ( Zhou *et al.*, 2023). The assembly was checked for contamination and corrected as described previously ( Howe *et al.*, 2021). Manual curation was performed using HiGlass ( Kerpedjiev *et al.*, 2018) and PretextView ( Harry, 2022). The mitochondrial genome was assembled using MitoHiFi ( Uliano-Silva *et al.*, 2023), which runs MitoFinder ( Allio *et al.*, 2020) or MITOS ( Bernt *et al.*, 2013) and uses these annotations to select the final mitochondrial contig and to ensure the general quality of the sequence.

### Evaluation of final assembly

The final assembly was post-processed and evaluated with the three Nextflow ( Di Tommaso *et al.*, 2017) DSL2 pipelines “sanger-tol/readmapping” ( Surana *et al.*, 2023a), “sanger-tol/genomenote” ( Surana *et al.*, 2023b), and “sanger-tol/blobtoolkit” ( Muffato *et al.*, 2024). The pipeline sanger-tol/readmapping aligns the Hi-C reads with bwa-mem2 ( Vasimuddin *et al.*, 2019) and combines the alignment files with SAMtools ( Danecek *et al.*, 2021). The sanger-tol/genomenote pipeline transforms the Hi-C alignments into a contact map with BEDTools ( Quinlan & Hall, 2010) and the Cooler tool suite ( Abdennur & Mirny, 2020), which is then visualised with HiGlass ( Kerpedjiev *et al.*, 2018). It also provides statistics about the assembly with the NCBI datasets ( Sayers *et al.*, 2024) report, computes *k*-mer completeness and QV consensus quality values with FastK and MerquryFK, and a completeness assessment with BUSCO ( Manni *et al.*, 2021).

The sanger-tol/blobtoolkit pipeline is a Nextflow port of the previous Snakemake Blobtoolkit pipeline ( Challis *et al.*, 2020). It aligns the PacBio reads with SAMtools and minimap2 ( Li, 2018) and generates coverage tracks for regions of fixed size. In parallel, it queries the GoaT database ( Challis *et al.*, 2023) to identify all matching BUSCO lineages to run BUSCO ( Manni *et al.*, 2021). For the three domain-level BUSCO lineage, the pipeline aligns the BUSCO genes to the Uniprot Reference Proteomes database ( Bateman *et al.*, 2023) with DIAMOND ( Buchfink *et al.*, 2021) blastp. The genome is also split into chunks according to the density of the BUSCO genes from the closest taxonomically lineage, and each chunk is aligned to the Uniprot Reference Proteomes database with DIAMOND blastx. Genome sequences that have no hit are then chunked with seqtk and aligned to the NT database with blastn ( Altschul *et al.*, 1990). All those outputs are combined with the blobtools suite into a blobdir for visualisation.

All three pipelines were developed using the nf-core tooling ( Ewels *et al.*, 2020), use MultiQC ( Ewels *et al.*, 2016), and make extensive use of the Conda package manager, the Bioconda initiative ( Grüning *et al.*, 2018), the Biocontainers infrastructure ( da Veiga Leprevost *et al.*, 2017), and the Docker ( Merkel, 2014) and Singularity ( Kurtzer *et al.*, 2017) containerisation solutions.

Table 3 contains a list of relevant software tool versions and sources.

**Table 3.** Software tools: versions and sources.

Software tool	Version	Source
BEDTools	2.30.0	https://github.com/arq5x/bedtools2
Blast	2.14.0	ftp://ftp.ncbi.nlm.nih.gov/blast/executables/blast+/
BlobToolKit	4.3.7	https://github.com/blobtoolkit/blobtoolkit
BUSCO	5.4.3 and 5.5.0	https://gitlab.com/ezlab/busco
bwa-mem2	2.2.1	https://github.com/bwa-mem2/bwa-mem2
Cooler	0.8.11	https://github.com/open2c/cooler
DIAMOND	2.1.8	https://github.com/bbuchfink/diamond
fasta_windows	0.2.4	https://github.com/tolkit/fasta_windows
FastK	427104ea91c78c3b8b8b49f1a7d6bbeaa869ba1c	https://github.com/thegenemyers/FASTK
GoaT CLI	0.2.5	https://github.com/genomehubs/goat-cli
Hifiasm	0.19.5-r587	https://github.com/chhylp123/hifiasm
HiGlass	44086069ee7d4d3f6f3f0012569789ec138f42b84 aa44357826c0b6753eb28de	https://github.com/higlass/higlass
MerquryFK	d00d98157618f4e8d1a9190026b19b471055b22e	https://github.com/thegenemyers/MERQURY.FK
MitoHiFi	3	https://github.com/marcelauliano/MitoHiFi
MultiQC	1.14, 1.17, and 1.18	https://github.com/MultiQC/MultiQC
NCBI Datasets	15.12.0	https://github.com/ncbi/datasets
Nextflow	23.04.0-5857	https://github.com/nextflow-io/nextflow
PretextView	0.2	https://github.com/wtsi-hpag/PretextView
purge_dups	1.2.5	https://github.com/dfguan/purge_dups
samtools	1.16.1, 1.17, and 1.18	https://github.com/samtools/samtools
sanger-tol/genomenote	1.1.1	https://github.com/sanger-tol/genomenote
sanger-tol/readmapping	1.2.1	https://github.com/sanger-tol/readmapping
Seqtk	1.3	https://github.com/lh3/seqtk
Singularity	3.9.0	https://github.com/sylabs/singularity
YaHS	1.2a.2	https://github.com/c-zhou/yahs

### Genome annotation

The Ensembl Genebuild annotation system ( Aken *et al.*, 2016) was used to generate annotation for the *Nesoenas mayeri* assembly (GCA_963082525.1) in Ensembl Rapid Release at the EBI. Annotation was created primarily through alignment of transcriptomic data to the genome, with gap filling via protein-to-genome alignments of a select set of proteins from UniProt ( UniProt Consortium, 2019).

### Wellcome Sanger Institute – Legal and Governance

The materials that have contributed to this genome note have been supplied by a Tree of Life collaborator. The Wellcome Sanger Institute employs a process whereby due diligence is carried out proportionate to the nature of the materials themselves, and the circumstances under which they have been/are to be collected and provided for use. The purpose of this is to address and mitigate any potential legal and/or ethical implications of receipt and use of the materials as part of the research project, and to ensure that in doing so we align with best practice wherever possible.

The overarching areas of consideration are:

•   Ethical review of provenance and sourcing of the material

•   Legality of collection, transfer and use (national and international)

Each transfer of samples is undertaken according to a Research Collaboration Agreement or Material Transfer Agreement entered into by the Tree of Life collaborator, Genome Research Limited (operating as the Wellcome Sanger Institute) and in some circumstances other Tree of Life collaborators.

## Data Availability

European Nucleotide Archive: *Nesoenas mayeri* (Pink Pigeon). Accession number PRJEB64092; https://identifiers.org/ena.embl/PRJEB64092 ( Wellcome Sanger Institute, 2023). The genome sequence is released openly for reuse. The *Nesoenas mayeri* genome sequencing initiative is part of the Vertebrate Genomes Project. All raw sequence data and the assembly have been deposited in INSDC databases. Raw data and assembly accession identifiers are reported in Table 1.

## Author information

Members of the Wellcome Sanger Institute Tree of Life Management, Samples and Laboratory team are listed here: https://doi.org/10.5281/zenodo.10066175.

Members of Wellcome Sanger Institute Scientific Operations: Sequencing Operations are listed here: https://doi.org/10.5281/zenodo.10043364.

Members of the Wellcome Sanger Institute Tree of Life Core Informatics team are listed here: https://doi.org/10.5281/zenodo.10066637.

Members of the Tree of Life Core Informatics collective are listed here: https://doi.org/10.5281/zenodo.5013541.
