# Supplementary material for: The genome sequence of the Pink Pigeon, Nesoenas mayeri (Prévost, 1843)
Source: Wellcome Open Res. 2025 Oct 10;9:336. Originally published 2024 Jun 24. [Version 2] doi: 10.12688/wellcomeopenres.22471.2 (PMC12635523; doi:10.12688/wellcomeopenres.22471.2)
Supplement: Peer review history

# Reviewer report: Reviewer response for version 2

**DOI:** 10.21956/wellcomeopenres.27572.r135907

**Published:** 2025-11-20

**Authors:** Rauri C.K. Bowie

**Affiliations:** 1 University of California Berkeley, Berkeley, USA

This is a well-written and informative description of a high-quality reference genome for the Pink Pigeon. I have reviewed the revised version of the manuscript and have no further comments. I find the authors’ responses to the previous review comments to be thorough and satisfactory.

 This genome will greatly enhance the ability to align historical genomes from museum specimens of Pink Pigeons collected over the past 150 years, revealing novel mutational variation that—through emerging technologies—may facilitate the genetic rescue of the current bottlenecked population.

Are sufficient details of methods and materials provided to allow replication by others?

Yes

Is the rationale for creating the dataset(s) clearly described?

Yes

Are the datasets clearly presented in a useable and accessible format?

Yes

Are the protocols appropriate and is the work technically sound?

Yes

Reviewer Expertise:

Evolutionary Biology, Genomics, Birds

I confirm that I have read this submission and believe that I have an appropriate level of expertise to confirm that it is of an acceptable scientific standard.

# Reviewer report: Reviewer response for version 2

**DOI:** 10.21956/wellcomeopenres.27572.r135740

**Published:** 2025-10-14

**Authors:** Thomas Liehr

**Affiliations:** 1 Friedrich Schiller University, Jena, Germany

Thanks for replies.

 point 1 - accepted

 point 2 - well - without looking at chromosomal level one can not be sure if alignments are correct - the previous comment was less for micro- but macrochromosomes - so question - how do authors make sure the macrochromosome aligmnements are correct?

Are sufficient details of methods and materials provided to allow replication by others?

Yes

Is the rationale for creating the dataset(s) clearly described?

Yes

Are the datasets clearly presented in a useable and accessible format?

No

Are the protocols appropriate and is the work technically sound?

Yes

Reviewer Expertise:

chromosomal evolution in different animals including mammals, fishes, birds and others

I confirm that I have read this submission and believe that I have an appropriate level of expertise to confirm that it is of an acceptable scientific standard, however I have significant reservations, as outlined above.

# Reviewer report: Reviewer response for version 1

**DOI:** 10.21956/wellcomeopenres.24759.r95300

**Published:** 2024-09-05

**Authors:** Xiaoping Zhou

**Affiliations:** 1 Xiamen University, Xiamen, China

This manuscript presents a high-quality chromosome-scale assembly for the Pink Pigeon.

**I just have two minor comments:**

 1) In page 4, "and the assembly has a BUSCO v completeness of 97.3% (single = 96.9%, duplicated = 0.4%), using the vertebrata_odb10 reference set (n = 8,338)".  The letter v after BUSCO should be deleted.

 2) The vertebrata_odb10 was used in sentence mentioned above and also in the notes to Table 1, but aves_odb10 was used in Figure 2.

Are sufficient details of methods and materials provided to allow replication by others?

Yes

Is the rationale for creating the dataset(s) clearly described?

Yes

Are the datasets clearly presented in a useable and accessible format?

Yes

Are the protocols appropriate and is the work technically sound?

Yes

Reviewer Expertise:

molecular ecology

I confirm that I have read this submission and believe that I have an appropriate level of expertise to confirm that it is of an acceptable scientific standard.

# Reviewer report: Reviewer response for version 1

**DOI:** 10.21956/wellcomeopenres.24759.r93552

**Published:** 2024-08-16

**Authors:** Thomas Liehr

**Affiliations:** 1 Friedrich Schiller University, Jena, Germany

The paper is of interest as a rare pigeon species is sequenced.

**What is lacking is:**

- The alignment/ comparison of it with at least one other pigeon species 
- Comments on restrictions of this study - like that the data is only really useful if it is aligned with (molecular) cytogenetic data; only then the real chromosome number of this species can be found out and also which 'pseudochromosomes' indeed form a common real chromosome. Also only due to that data chromosomal evolution can be understood. 

Are sufficient details of methods and materials provided to allow replication by others?

Yes

Is the rationale for creating the dataset(s) clearly described?

Yes

Are the datasets clearly presented in a useable and accessible format?

No

Are the protocols appropriate and is the work technically sound?

Yes

Reviewer Expertise:

chromosomal evolution in different animals including mammals, fishes, birds and others

I confirm that I have read this submission and believe that I have an appropriate level of expertise to state that I do not consider it to be of an acceptable scientific standard, for reasons outlined above.

# Response

**Published:** 2025-09-23

**Authors:** Tree of Life Team Sanger

**Affiliations:** Wellcome Trust Sanger Institute, UK

We thank the reviewer for their comments.

1. **Comparative analyses with other pigeon species**
  We note the request for alignment and comparison with other pigeon genomes. However, this article is submitted as a *data note*, whose primary purpose is to present a high-quality genome assembly as a resource for the community. Data notes typically do not include detailed comparative analyses, which are more appropriately the subject of follow-up studies that build upon this dataset.
2. **Cytogenetic data and chromosome validation**
  The reviewer highlights that full understanding of avian chromosome organisation requires molecular cytogenetic data. We agree that cytogenetics provides valuable complementary insights. In this work, however, chromosomes have been curated by an experienced genome finishing team using Hi-C data, which is the standard approach for achieving chromosome-level assemblies in birds and many other taxa. While microchromosomes are indeed challenging to resolve, the assembly and curation processes applied here represent the current best practice in the field, and the resource will facilitate future cytogenetic and evolutionary studies.

We believe this genome assembly is of significant value in its own right, especially given the rarity of the species. It will serve as a reference point for future work on chromosomal evolution, cytogenetics, and comparative genomics within pigeons and other birds.

 The Tree of Life team
